# PD-L1 Dysregulation in COVID-19 Patients

**DOI:** 10.3389/fimmu.2021.695242

**Published:** 2021-06-07

**Authors:** Francesco Sabbatino, Valeria Conti, Gianluigi Franci, Carmine Sellitto, Valentina Manzo, Pasquale Pagliano, Emanuela De Bellis, Alfonso Masullo, Francesco Antonio Salzano, Alessandro Caputo, Ilaria Peluso, Pio Zeppa, Giosuè Scognamiglio, Giuseppe Greco, Carla Zannella, Michele Ciccarelli, Claudia Cicala, Carmine Vecchione, Amelia Filippelli, Stefano Pepe

**Affiliations:** ^1^ Department of Medicine, Surgery and Dentistry, “Scuola Medica Salernitana”, University of Salerno, Baronissi (SA), Italy; ^2^ Oncology Unit, San Giovanni di Dio e Ruggi D’Aragona University Hospital, Salerno, Italy; ^3^ Pharmacology Unit, San Giovanni di Dio e Ruggi D’Aragona University Hospital, Salerno, Italy; ^4^ Clinical Pathology and Microbiology Unit, San Giovanni di Dio e Ruggi D’Aragona University Hospital, Salerno, Italy; ^5^ Infectious Disease Unit, San Giovanni di Dio e Ruggi D’Aragona University Hospital, Salerno, Italy; ^6^ Otolaryngology Unit, San Giovanni di Dio e Ruggi D’Aragona University Hospital, Salerno, Italy; ^7^ Pathology Unit, San Giovanni di Dio e Ruggi D’Aragona University Hospital, Salerno, Italy; ^8^ Hematology Unit, AORN Cardarelli Hospital, Naples, Italy; ^9^ Pathology Unit, Istituto Nazionale Tumori, IRCSS, “Fondazione G Pascale”, Naples, Italy; ^10^ Section of Microbiology and Virology, University Hospital “Luigi Vanvitelli”, Naples, Italy; ^11^ Department of Experimental Medicine, University of Campania “Luigi Vanvitelli”, Naples, Italy; ^12^ Cardiology Unit, San Giovanni di Dio e Ruggi D’Aragona University Hospital, Salerno, Italy; ^13^ Laboratory of Immunoregulation, National Institute of Allergy and Infectious Diseases, Bethesda, MD, United States; ^14^ Vascular Pathophysiology Unit, IRCCS Neuromed, Pozzilli, Italy

**Keywords:** SARS-CoV-2, PD-L1, immune checkpoint molecules, innate immune response, adaptive immune response, COVID-19, ARDS, prognosis

## Abstract

The COVID-19 pandemic has reached direct and indirect medical and social consequences with a subset of patients who rapidly worsen and die from severe-critical manifestations. As a result, there is still an urgent need to identify prognostic biomarkers and effective therapeutic approaches. Severe-critical manifestations of COVID-19 are caused by a dysregulated immune response. Immune checkpoint molecules such as Programmed death-1 (PD-1) and its ligand programmed death-ligand 1 (PD-L1) play an important role in regulating the host immune response and several lines of evidence underly the role of PD-1 modulation in COVID-19. Here, by analyzing blood sample collection from both hospitalized COVID-19 patients and healthy donors, as well as levels of PD-L1 RNA expression in a variety of model systems of SARS-CoV-2, including *in vitro* tissue cultures, ex-vivo infections of primary epithelial cells and biological samples obtained from tissue biopsies and blood sample collection of COVID-19 and healthy individuals, we demonstrate that serum levels of PD-L1 have a prognostic role in COVID-19 patients and that PD-L1 dysregulation is associated to COVID-19 pathogenesis. Specifically, PD-L1 upregulation is induced by SARS-CoV-2 in infected epithelial cells and is dysregulated in several types of immune cells of COVID-19 patients including monocytes, neutrophils, gamma delta T cells and CD4+ T cells. These results have clinical significance since highlighted the potential role of PD-1/PD-L1 axis in COVID-19, suggest a prognostic role of PD-L1 and provide a further rationale to implement novel clinical studies in COVID-19 patients with PD-1/PD-L1 inhibitors.

## Introduction

COVID-19 pandemic caused by severe acute respiratory syndrome coronavirus-2 (SARS-CoV-2) holds the world in thrall since early March 2020. COVID-19 manifests a spectrum of signs and symptoms from mild illness to acute pneumonia. Unfortunately, a considerable percentage of patients rapidly worse to acute respiratory distress syndrome (ARDS) requiring intensive care (1, 2).

Understanding the link between patients’ immune features and disease severity represents a crucial step in the war against this pandemic. Severe-critical manifestations of COVID-19 are caused by a dysregulated immune response in which the adaptive immune system, ruled by T and B lymphocytes, plays a fundamental role (3).

T cells fulfill specific antiviral actions inside a complex inflammatory milieu influencing both the cellular and humoral immunity (4). During a chronic infection, including COVID-19, these cells are either eliminated or become dysfunctional until exhaustion (4, 5). The reduction of T cell count as well as increased levels of biochemical parameters of inflammation correlate with a poor prognosis in COVID-19 patients and have been proposed to set up a more aggressive treatment in order to avoid a sudden worsening of clinical conditions (5).

Immune checkpoint molecules, including Programmed death-1 (PD-1) and its ligand programmed death-ligand 1 (PD-L1), play an important role in innate and especially adaptive immune response by serving as modulators. The PD-1/PD-L1 axis is a major contributor among the checkpoint molecules in maintaining the delicate balance between immune response and immune-mediated cellular damage during inflammation (6). Such signaling is involved in several types of infections such as in human immunodeficiency virus (HIV) and hepatitis C virus (HCV) (7–9).

PD-1 calibrates qualitatively and quantitatively T cell responses against cancer (10) and its role during both acute and chronic infection has been quite characterized (9).

Recently, it has been reported that in severe and critical COVID-19 patients T cells, shifting from a status of hyperactivation to one of exhaustion, express increased levels of PD-1 (5, 11).

In contrast, there are few and inconclusive data about the significance of PD-L1 dysregulation during SARS-CoV-2 infection and no data are currently available on the role of soluble PD-L1 (sPD-L1) in COVID-19 patients with a different grade of disease severity and prognosis.

The present study aimed to investigate the role of PD-L1 in COVID-19 prognosis and pathogenesis.

## Materials and Methods

### Patient Characteristics and Biochemical Parameters

Patients with a confirmed diagnosis of COVID-19 and healthy donors from “San Giovanni di Dio e Ruggi D’Aragona” University Hospital were recruited from October 2020 to January 2021. All patients with COVID-19 pneumonia diagnosed based on characteristic radiological findings and a positive naso-pharyngeal swab for SARS-CoV-2 RNA, were evaluated to be included in the study. Patient selection was performed based on: (i) age >18 years; (ii) characteristic infiltrates observed by a chest CT scan; (iii) positive nasal swab for SARS-CoV-2-RNA at the time of hospital admission; (iv) informed consent for blood sample analysis. All participants were Caucasians. All of them signed informed consent. The study was approved by the local ethics committee (n.30_r.p.s.o./2020), in accordance with the Declaration of Helsinki and its amendments and was performed without interfering with normal clinical practice.

Demographic (age and gender) and pathological [comorbidities, diagnosis of COVID-19 associated pneumonia, need of high-flow oxygen therapy, length of hospital stay (LOS), time length of negativizaton to SARS-CoV-2, therapy, death or hospital discharge] data were retrieved from clinical records. Biochemical [number of peripheral blood cells (neutrophils, lymphocytes and platelets), lactate dehydrogenase (LDH), erythrocyte sedimentation rate (ESR), C-reactive protein (CRP) and fibrinogen] and arterial oxygen partial pressure/fractional inspired oxygen (PaO_2_/FIO_2_) parameters were collected as part of the standard workup at the Clinical Pathology Unit of “San Giovanni di Dio e Ruggi D’Aragona” University Hospital.

### Blood Sample Collection and Measurement of Soluble PD-L1

Peripheral blood samples were collected from each subject during routine venipuncture within 6 days from the admission to the hospital. Serum samples were isolated by centrifugation at 1000×g for 15 minutes (min) at 4°C and immediately stored at −80°C until analysis. Levels of soluble PD-L1 (sPD-L1) were determined by enzyme-linked immunosorbent assay (ELISA) according to the manufacturer’s instruction (Elabscience Biotechnology Co. Ltd, Wuhan, China). The optical density (OD) was measured spectrophotometrically using a plate reader (TECAN^®^ infinite 200 PRO) at a wavelength of 450 nm. Each sample was tested in duplicate. The sPD-L1 level was determined using a standard curve. The minimum detectable level of sPD-L1 was 0.10 ng/mL and the detection range was 0.16-10.0 ng/mL. The intra-assay and inter-assay coefficients of variation were below 10%. Data are expressed as the mean ± SD of three independent experiments.

### Tissue Sample and Immunohistochemical Staining of PD-L1

Formalin-fixed paraffin-embedded (FFPE) specimen of bronchial aspirate was obtained from patient # COVID-22 followed at “San Giovanni di Dio e Ruggi D’Aragona” University Hospital. The patient has consented for tissue acquisition per institutional review board-approved protocol. The patient signed informed consent. FFPE tissue sections (4 μm) from the bronchial aspirate sample were used as substrates in immunohistochemical (IHC) reactions. The PD-L1-specific monoclonal antibody (mAb) SP263 and the rabbit mAb IgG, utilized as an isotype control for PD-L1 staining, were purchased from VENTANA. The staining with SP263 mAb was performed on Ventana BenchMark XT automated IHC Stainer (VENTANA) utilizing OptiView DAB IHC Detection Kit (VENTANA). The staining intensity and percentage of stained cells were reviewed and enumerated by an experienced pathologist (PZ) who did not know the patient characteristics and clinical outcome. Staining with PD-L1-specific mAb was performed according to the manufacturers’ instructions and was scored by counting both the number of epithelial and immune stained cells in four high-powered fields.

### RNA-seq of PD-L1

RNA-seq profiles used to assess PD-L1 gene profiles in human cells and lung biopsies were collected from Gene Expression Omnibus (GEO) (12) accession GSE147507, GSE148729 and GSE56192. The cell lines used were human lung adenocarcinoma Calu-3, normal human bronchial epithelial (NHBE) and human adenocarcinoma alveolar basal epithelial A549 cell lines. A549, Calu3, NHBE cells were incubated at indicated times and indicated viral load (MOI) with SARS-CoV-2 and SARS-CoV-1 (Calu3). In addition, A549 cells were transduced with an expression vector encoding the human ACE2 protein and incubated for 24 hours with SARS-CoV-2, at different MOI and treated with ruxolitinib (500 nM), a JAK1 and JAK2 kinase inhibitor. Cells incubated with vector but without the virus (mock) and untreated cells were used as controls. Lung biopsies were derived from a deceased male COVID-19 patient (age 74) or uninfected patients [one male (age 72) and one female (age 60)]. Expression levels of PD-L1 RNA were the counts of reads aligned to the genome and expressed as counts per million. Values were extracted from supplemental data from GSE147507 and GSE148729. The vendors analyzed the sequencing libraries on the Illumina NextSeq 500. They then aligned the reads using the RNA-Seq Alignment app on Basespace (Illumina, CA).

RNA-seq profiles of immune cells were collected from the Immunological Genome Project (13) using the Skyline RNA-seq tool. Eight peripheral blood samples from 7 hospitalized patients with RT-PCR-confirmed SARS-CoV-2 infection and 6 healthy controls were included. Samples were collected between 2 and 16 days after the onset of symptoms. Four of 8 COVID-19 samples were collected from ventilated patients diagnosed with acute respiratory distress syndrome (ARDS); the remaining 4 samples were from less critically ill patients. One patient was sampled twice, initially at 9 days after symptom onset while admitted and requiring supplemental oxygen but not ventilated, and again at 11 days after symptom onset following intubation. Thresholds on expression values were derived for each platform by one of two distribution-based approaches. For platforms with well-defined negative control probe sets, the threshold for greater-than-chance expression was defined as expression values greater than or equal to the 95% quantile of expression values in the negative controls.

### Statistical Analysis

Statistical analysis was performed with the Stata Statistical Software, Version 13.0 (StataCorp, LP). Correlations between sPD-L1 levels, pathological and clinical characteristics of COVID-19 patients were analyzed by Spearman rank correlation test. Differences in the expression levels of variables according to pathological and clinical characteristics were analyzed using the Mann–Whitney U test. The correlation of sPD-L1 levels with the number of deaths was analyzed by Fisher exact test. For RNA-seq analysis, gene counts were normalized using the EdgeR package, Bioconductor (14) which considers that most genes are invariant between experiments. PD-L1 RNA levels result from similar processing of the reads involving the DESeq2 package (15). Statistical analyses were performed using Prism 9.0.0 (16). Pairwise comparisons were generated for all cell types with the control and PD-L1 was identified whose expression was significantly different for each cell type. p<0.05 was considered to be statistically significant. All tests used were two-tailed.

## Results

### Patient Characteristics

A total of 31 patients with a confirmed diagnosis of COVID-19 from “San Giovanni di Dio e Ruggi D’Aragona University Hospital” were recruited from October 2020 to January 2021. All baseline medical record information including clinical characteristics and laboratory data are shown in **Table 1**. Among the 31 patients, 4 (12.9%), 15 (48.4%) and 12 (38.7%) were mild, moderate-severe and critical cases, respectively. Twenty-nine (93.55%) had the diagnosis of COVID-19 associated pneumonia and 23 (74.19%) required high-flow oxygen therapy. Twenty patients (64.5%) suffered from chronic diseases including hypertension (51.6%), diabetes (25.8%), dyslipidemia (19.3%), cardiovascular disease (12.9%), immune disorder (9.7%), chronic pulmonary disease (9.7%) and chronic kidney disease (6.4%) (**Supplementary Table 1** **)**. The mean hospital LOS was 42.48 days (range, 19-83) while the meantime length of negativization for SARS-CoV-2 was 38.43 days (range, 15-75). Most of patients were treated with corticosteroids (90.32%), low molecular weight heparin (LMWH) (90.32%), azithromycin (83.87%), ceftriaxone (29.03%), ruxolitinib (6.45%), eculizumab (6.45%) and tocilizumab (6.45%) (**Supplementary Table 2** **)**. Five patients (16.13%) died from ARDS while 26 patients (83.87%) were discharged.

**Table 1:** Patient characteristics.

**Age**	**65.2 years** (31–90)
**Sex:**	
Male	**14** (45.16%)
Female	**17** (54.84%)
**Mean number of peripheral blood cells:**	
Neutrophils	**5525.6 x10^3^/µL** (range, 1100-9560)
Lymphocytes	**834.4 x10^3^/µL** (range, 2.07-3360)
Platelets	**266767.7 x10^3^/µL** (range, 6000-539000)
**LDH**	**528.6 U/l** (range, 160-2191)
**ESR**	**52.64 mm** (range, 20-80)
**CRP**	**5.11 mg/dl** (range, 0.05-18.69)
**Fibrinogen**	**464.1 mg%** (range, 126-775)
**COVID-19 associated pneumonia:**	
Presence	**29** (93.55%)
Absence	**2** (6.45%)
**High-flow oxygen therapy**	
Yes	**23** (74.19%)
Not	**8** (35.81%)
**Chronic diseases:**	
Hypertension	**16** (51.6%)
Diabetes	**8** (25.8%)
Dysplipidemia	**6** (19.3%)
Cardiovascular disease	**4** (12.9%)
Immune disorder	**3** (9.7%)
Chronic pulmonary disease	**3** (9.7%)
Chronic kidney disease	**2** (6.4%)
Absence	**9** (29.0%)
**Mean number of LOS**	**42.48 days** (range, 19-83)
**Mean time length of negativization**	**38.43 days** (range, 15-75)
**Therapy:**	
Corticosteroids	**29** (90.32%)
Low molecular weight heparin	**29** (90.32%)
Azithromycin	**27** (83.87%)
Ceftriaxone	**9** (29.03%)
Ruxolitinib	**2** (6.45%)
Eculizumab	**2** (6.45%)
Tocilzumab	**2** (6.45%)
**Nember of deaths**	**5** (16.12%)

### Correlation Between Pathological and Clinical Characteristics of COVID-19 Patients

Age of patients correlated with the number of peripheral lymphocytes (Spearman’s rho -0.3196, p=0.0852), serum CRP levels (Spearman’s rho 0.3749, p=0.0412), required high-flow oxygen therapy (Spearman’s rho 0.4621, p=0.0101), LOS (Spearman’s rho 0.5421, p=0.0020), time length of negativization for SARS-CoV-2 (Spearman’s rho 0.4002, p=0.0284) and the number of deaths (p=0.0223). The number of deaths was significantly higher in older patients [mean 79.2 years, (range, 61-90)] as compared to younger patients [mean 62.4, (range 31-84)].

The number of peripheral lymphocytes was negatively correlated with serum LDH and CRP levels (LDH: Spearman’s rho -0.4318, p=0.0153; CRP: Spearman’s rho -0.4764, p=0.0067), LOS (Spearman’s rho -0.4769, p=0.0067) and time length of negativization for SARS-CoV-2 (Spearman’s rho -0.3629, p=0.0448). Besides, serum LDH levels correlated with LOS (Spearman’s rho 0.4211, p=0.0183) and the number of deaths (p=0.0763).

Levels of ESR correlated with required high-flow oxygen therapy (p=0.0085), LOS (Spearman’s rho 0.5106, p=0.0621) and time length of negativization for SARS-CoV-2 (Spearman’s rho 0.5089, p=0.0631).

Lastly levels of CRP correlated with required high-flow oxygen therapy (p=0.0072), LOS (Spearman’s rho 0.5159, p=0.0030), time length of negativization for SARS-CoV-2 (Spearman’s rho 0.3825, p=0.0337) and the number of deaths (p=0.0052). As expected, increased LOS correlated with the number of deaths (p=0.0007).

### Serum Levels of sPD-L1 and Its Comparison in COVID-19 Patients With Different Clinicopathological Characteristics

**Figure 1:**
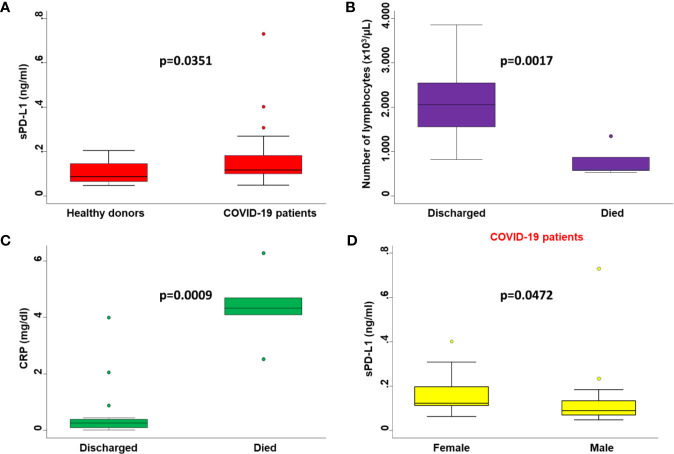
Comparisons between serum levels of biochemical parameters in patients with different clinicopathological characteristics. **(A)** Serum levels of sPD-L1 in COVID-19 patients were compared with those of healthy donors by Mann-Whitney U test. **(B)** The number of peripheral lymphocytes in COVID-19 patients who died or were discharged from the hospital were compared by the Mann-Whitney U test. **(C)** Serum levels of CRP in COVID-19 patients who died or were discharged from the hospital were compared by the Mann-Whitney U test. **(D)** Serum levels of sPD-L1 in male and female COVID-19 patients were compared by the Mann-Whitney U test. On each box, the central mark is the median, the edges of the box are the 25th and 75th percentiles, the whiskers extend to the most extreme data points not considered outliers, and outliers are plotted individually. p was considered significant if < 0.05.

We first evaluated and compared serum levels of sPD-L1 from all 31 COVID-19 patients within 6 days [mean number 4.75 days (range, 3-6 days)] of the hospital admission with those of 24 healthy donors without SARS-CoV-2. The mean serum level of sPD-L1 in hospitalized COVID-19 patients and non-infected patients was 0.162 ng/ml (range, 0.0479-0.730) and 0.103 ng/m (range, 0.0472-0.204), respectively (**Figure 1A**). The serum levels of sPD-L1 were significantly higher in COVID-19 patients as compared to those of non-infected patients (p=0.0351).

We then correlated serum levels of sPD-L1 with blood sample biomarkers as well as with levels of PaO_2_/FIO_2_ at the same day of PD-L1 sample collection. The mean number of neutrophils, lymphocytes and platelets was 5579.5 x10^3^/µL (range, 1780-10900), 1888.4 x10^3^/µL (range, 560-3860) and 257466.7 x10^3^/µL (range, 81000-471000), respectively. The mean level of LDH, ESR, CRP and fibrinogen was 276.6 U/l (range, 121-979), 40.58 mm (range, 13-129), 1.292 mg/dl (range, 0.02-6.28) and 318.2 mg% (33-731), respectively. The mean level of PaO_2_/FIO_2_ was 320.11 mmHg (range, 142-571).

**Figure 2:**
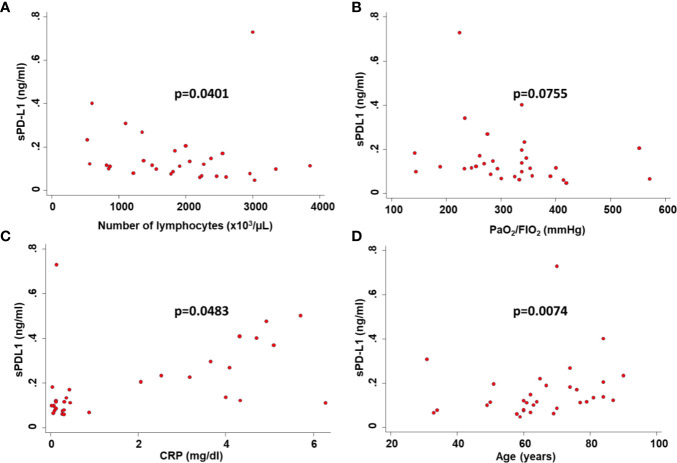
Correlation between serum levels of sPD-L1 and clinicopathological characteristics of COVID-19 patients. Serum levels of sPD-L1 in COVID-19 patients were correlated with the number of peripheral lymphocytes **(A)**, the level of PaO_2_/FIO_2_ **(B)**, the level of CRP **(C)** and the age of patients **(D)** by Spearman’s correlations. p was considered significant if < 0.05.

**Figure 3:**
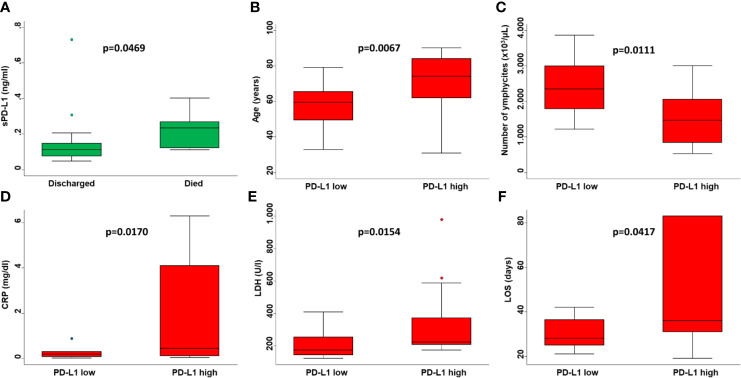
Prognostic value of sPD-L1 in COVID-19 patients. **(A)** Serum levels of sPD-L1 in COVID-19 patients who died or were discharged from the hospital were compared by Mann-Whitney U test. Age of patients **(B)**, number of lymphocytes **(C)**, levels of CRP **(D)**, levels of LDH **(E)** and LOS **(F)** grouped based on sPD-L1 levels were compared using the Mann-Whitney U test. On each box, the central mark is the median, the edges of the box are the 25th and 75th percentiles, the whiskers extend to the most extreme data points not considered outliers, and outliers are plotted individually. p was considered significant if < 0.05.

Consistent with baseline results, the number of lymphocytes (p=0.0017) (**Figure 1B**) and levels of CRP (p=0.0009) (**Figure 1C**) at the time of PD-L1 measurement significantly correlated with the number of deaths from COVID-19. In COVID-19 patients serum levels of sPD-L1 were significantly and negatively correlated with both total number of lymphocytes (Spearman’s rho -0.3353, p=0.0401) (**Figure 2A**) and levels of PaO_2_/FIO_2_ (Spearman’s rho -0.3274, p=0.0755) (**Figure 2B**). In contrast serum levels of sPD-L1 significantly and positively correlated with levels of CRP (Spearman’s rho 0.3988, p=0.0483) (**Figure 2C**). Patients displaying higher levels of sPD-L1 also displayed a low number of lymphocytes and PaO_2_/FIO_2_ as well as a high level of CRP. In addition, serum levels of sPD-L1 significantly correlated with the age and sex of patients. Specifically female patients displayed higher levels of sPD-L1 as compared to males (p=0.0472) (**Figure 1D**). Older patients displayed a higher level of sPD-L1 as compared to that of younger patients (Spearman’s rho 0.4789, p=0.0074) (**Figure 2D**). More importantly serum levels of sPD-L1 significantly correlated with the prognosis of COVID-19 patients (p=0.0469). Specifically, levels of PD-L1 were higher in all those patients who later died during the hospitalization (mean 0.227, range 0.111-0.402) as compared to patients who were discharged (mean 0.142, range 0.0479-0.730) (**Figure 3A**). Patients with high levels of PD-L1 were characterized by old age (p=0.0067) (**Figure 3B**), low number of lymphocytes (p=0.0111) (**Figure 3C**), high levels of CRP (p=0.0170) (**Figure 3D**), high levels of LDH (p=0.0154) (**Figure 3E**), high LOS (p=0.0417) (**Figure 3F**) and high number of deaths (p=0.048). Noteworthy no correlations between serum levels of sPD-L1 with patient comorbidities as well as with patient therapies (**Supplementary Tables 1** and **2**).

### PD-L1 Up-Regulation in SARS-CoV-2 Infected Cells

We characterized PD-L1 RNA levels in a variety of model systems of SARS-CoV-2 including *in vitro* tissue cultures, *ex-vivo* infections of primary epithelial cells and biological samples obtained from tissue biopsies of COVID-19 patients. RNA-seq results from the GEO database were downloaded.

**Figure 4:**
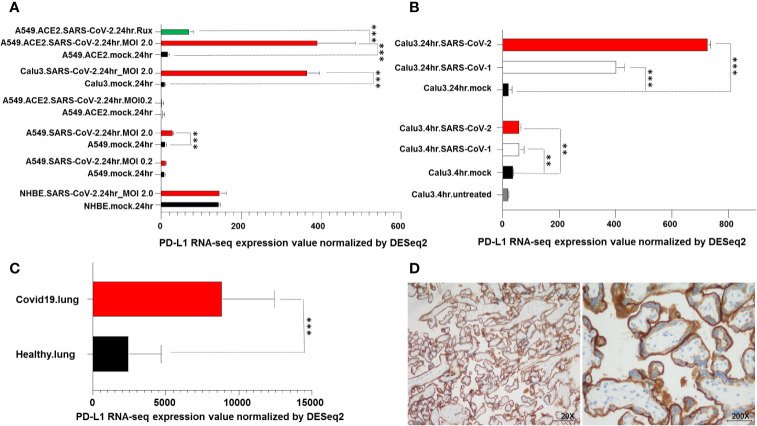
PD-L1 upregulation in SARS-CoV-2 infected cells and in COVID-19 patient lung biopsies. **(A)** RNA levels of PD-L1 in A549, Calu3, NHBE cells, lung specimens derived from deceased COVID-19 and uninfected patients were downloaded from the GEO database and analyzed. A549, Calu3, NHBE cells were incubated for 24 hours with SARS-CoV-2 (A549.SARS-CoV2, Calu3.SARS-CoV2 and NHBE.SARS-CoV2) at different viral load (MOI). A549 cells were transduced with an expression vector encoding the human ACE2 protein (A549.ACE2), incubated for 24 hours with Sars-CoV-2 (A549.ACE2.SARS-CoV2) at different viral load (MOI) and treated with ruxolitinib (500 nM) (A549.ACE2.SARS-CoV2.Rux). Cells incubated with vector but without the virus (mock) were used as a control (A549.mock, Calu3.mock, NHBE.mock, A549.ACE2.mock). **(B)** Calu3 cells were incubated with SARS-CoV-2 or SARS-CoV-1 (Calu3.SARS-CoV2 and Calu3.SARS-CoV1) at different time points. Cells non incubated (Calu3.untreated) or incubated with vector but without the virus (Calu3.mock) were used as controls. **(C)** Lung specimens derived from COVID-19 patients were considered and compared to samples of uninfected human lung biopsies. RNA levels of PD-L1 were compared using Pairwise comparisons. ** and *** indicate p < 0.001 and < 0.01, respectively. **(D)** Representative staining patterns of FFPE bronchial aspirate with PD-L1-specific mAb SP263 of a COVID-19 patient. IgG was used as an isotype control for PD-L1 staining (data not shown). Magnification is indicated.

Calu3 and NHBE cell lines were permissive to SARS-CoV-2. In Calu3 cells, following incubation with SARS-CoV-2, the levels of PD-L1 were significantly (P<0.001) increased as compared to those of mock cells (**Figure 4A**). In NHBE cells SARS-CoV-2 and mock incubation did not change the transcriptional levels of PD-L1. A549 cells were relatively non-permissive to SARS-CoV-2 (**Figure 4A**). However following transduction with an expression vector encoding the human ACE2 protein they became permissive to SARS-CoV-2. In both A549 cells and ACE2-transduced A549 cells SARS-CoV-2 incubation at low MOI (0.2) did not change the PD-L1 levels as compared to that of mock cells. However, a dramatic increase in viral load (MOI 2) was significantly (P< 0.001) associated with an increase in PD-L1 RNA-seq levels in A549 cells and even more to a greater extent in ACE2-transduced A549 cells. Noteworthy in these cells transcriptional levels of PD-L1 were abolished after ruxolitinib treatment (**Figure 4A**).

Analysis of PD-L1 RNA levels following incubation with SARS-CoV-2 at different time points demonstrated that PD-L1 levels were significantly (P<0.01) increased following 4-hour incubation and even more to a greater extent following 24-hour incubation with both SARS-CoV-1 and SARS-CoV-2 as compared to non-infected cells (**Figure 4B**).

Analysis of PD-L1 RNA levels obtained from post mortem lung biopsies of COVID-19 patients and lung tissue biopsies from healthy uninfected individuals demonstrated a significant (P<0.001) increase in PD-L1 transcript levels in COVID-19 patients as compared to healthy subjects (**Figure 4C**). Lastly, an IHC staining of a bronchial aspirate obtained from a COVID-19 patient analyzed for sPD-L1 levels (76 years old, female, requiring high-flow oxygen therapy and discharged following 83 days of hospitalization) demonstrated a moderate PD-L1 expression on lung epithelium cells (**Figure 4D**).

### PD-L1 Dysregulation in Immune Cells of COVID-19 Patients

**Figure 5:**
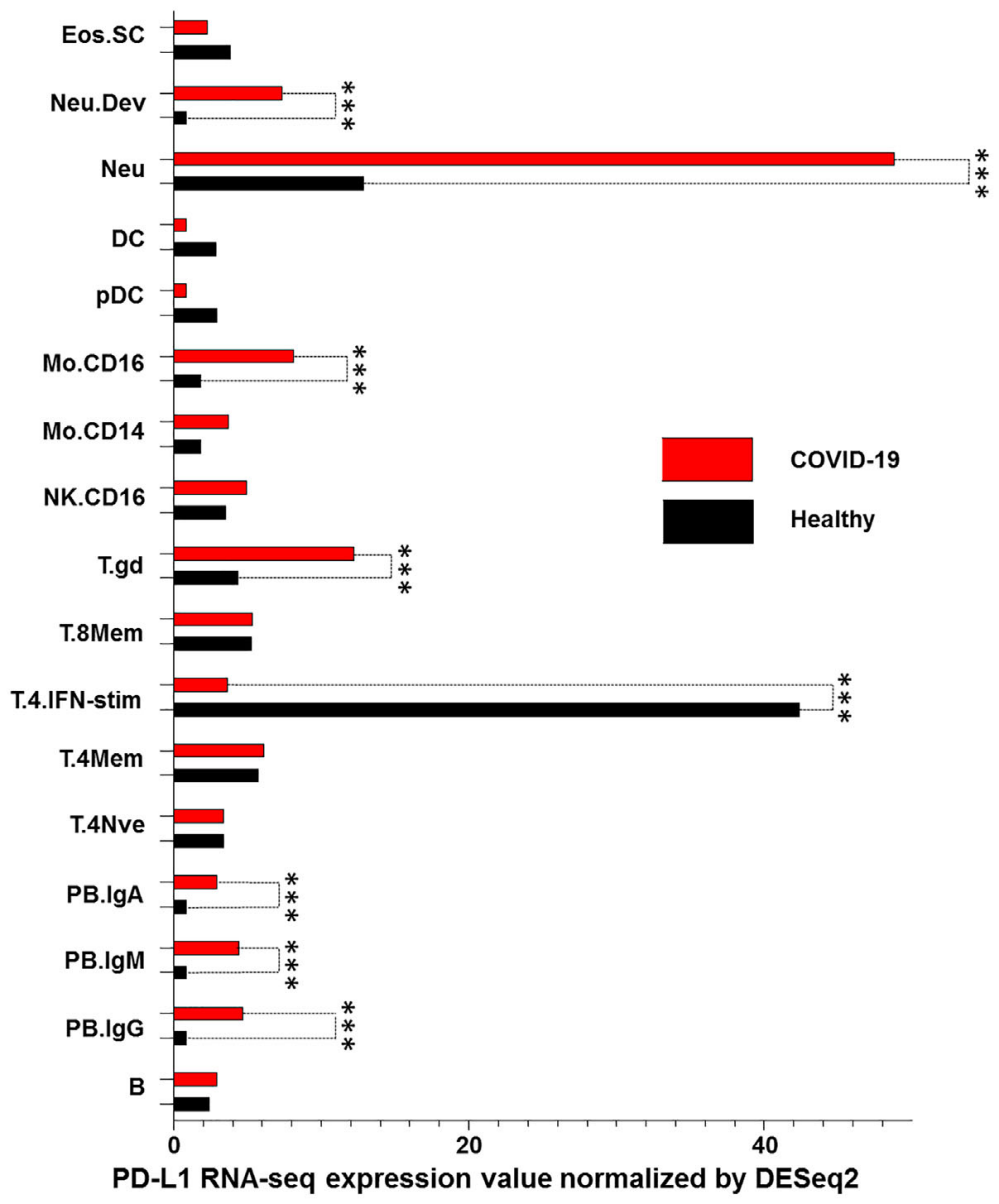
PD-L1 dysregulation in PBMC of COVID-19 patients. RNA levels of PD-L1 in PBMCs derived from COVID-19 patients and healthy donors were down-loaded from the Immunological Genome Project database and analyzed. PBMC were characterized as stem cells and eosinophils (Eos.SC), developing neutrophils (Neu.Dev), neutrophils (Neu), dendritic cells (DC), plasmacytoid dendritic cells (pDC), monocytes CD16+ and CD14+ (Mo.CD16 and Mo.CD14), natural killer cells (NK), gamma delta T cells (T.gd), CD8+ memory T cells (T.8Mem), CD4+ interferon-stimulated T cells (T.4.IFN-stim), CD4+ memory T cells (T.4Mem), CD4+ naive T cells (T.4Nve), IgA+ plasmablasts (PB.IgA), IgM+ plasmablasts (PB.IgM), IgG+ plasmablasts (PB.IgG) and B cells (B). RNA levels of PD-L1 for all cell types with the control were compared using Pairwise comparisons. *** indicate p < 0.001.

We lastly characterized PD-L1 RNA levels in peripheral blood mononuclear cells (PBMC) obtained from COVID-19 patients and compared them to those obtained from healthy donors. RNA-seq results from the Immunological Genome Project database (13) were downloaded. As expected, there was an increase in PD-L1 RNA levels in IgG, IgM and IgA plasmablasts in COVID-19 patients as compared to healthy donors. However, a significant increase in PD-L1 levels was also detected in neutrophils, monocytes (CD16+) and gamma delta T cells of COVID-19 patients (p<0.001) as compared to healthy donors. In addition, a significant decrease of PD-L1 levels (p<0.001) was detected in CD4+ T cells stimulated with interferon in COVID-19 patients as compared to healthy donors (**Figure 5**).

## Discussion

The COVID-19 pandemic has far reaching direct and indirect medical and social consequences. So far, there is still an unmet clinical need in defining patient prognosis and effective therapeutic approaches (17). Several parameters have been proposed to define COVID-19 patient prognosis. Biochemical parameters such as the number of lymphocytes and levels of CRP are shown to be effective prognostic biomarkers (18). Others, such as LDH, SAO_2_, PaO_2_/FIO_2_ and radiological findings, have been shown in some cases to contribute to defining COVID-19 diagnosis and prognosis (19, 20). The data we have shown are in line with these findings since the number of lymphocytes and CRP levels as well as LDH and PaO_2_/FIO_2_ correlated with patient prognosis assessed by the LOS and number of deaths from COVID-19. By corroborating the information in the literature, these results validate the representativeness of the patient population analyzed despite its limited number (21). In the present study, we have focused our analysis on characterizing the potential significance of sPD-L1 as a biomarker of patient prognosis. Our data demonstrate that PD-L1 might be useful to stratify COVID-19 patient prognosis as high levels of sPD-L1 correlated with validated prognostic biomarkers especially lymphopenia and high levels of CRP as well as with an increase of LOS and mortality rate. The levels of sPD-L1 were assessed within the first 6 days of hospital admission and were not later evaluated during the hospitalization of COVID-19 patients. Whether sPD-L1 levels still increase in those patients which are more likely to death from COVID-19 as compared to those discharged should be further investigated.

An increase in sPD-L1 is likely to reflect dysregulation of PD-1/PD-L1 axis in the host immune response of COVID-19 patients with a poor prognosis. The SARS-CoV-2 induces an extensive array of defense mechanisms in the host (22). Innate immunity tries to block or inhibits initial infection to protect the cells, or to eliminate virus-infected cells, and occurs well before the onset of an adaptive immune response. Innate immunity generally slows rather than stops a viral infection, allowing time for the adaptive immune response to begin. Antibody and T-cell-mediated immunity are the two major players of the adaptive immune response to viral infection. Antibodies usually bind to free viral particles, blocking infection of the host cell. In contrast, T cells act principally by recognizing and destroying virus-infected cells. In innate and especially adaptive immune responses, checkpoint molecules play an important role in maintaining the delicate balance of stimulating or inhibiting immune cell activation or inducing phenomena of self-tolerance or autoimmunity (23). The PD-1/PD-L1 axis is one of the major components of the checkpoint molecule family. Several lines of evidence indicate that the PD-1/PD-L1 axis might play a role in regulating the host immune response to SARS-CoV-2 as well as in COVID-19 pathogenesis (24–28). T cells, with CD8+ cytotoxic T cells (CTLs) capable of secreting an array of molecules to eradicate viruses from the host, are major players in SARS-CoV-2 clearance. At the same time, CD4+ helper T cells can assist cytotoxic T cells and B cells and enhance their ability to clear pathogens. However, persistent stimulation by the virus may induce T cell function reduction and exhaustion, leading to loss of T cell-related cytokine production. T cell exhaustion is defined by sustained expression of inhibitory receptors, and a transcriptional state distinct from that of functional effector or memory T cells (29). Diao et al. have recently shown that in COVID-19 patients, especially those critically ill, besides a negative correlation between T cell count (CD8+ and CD4+) and patient prognosis, both T cell types showed an exhaustive phenotype because of an increased expression of PD-1 and TIM-3. Moreover, an increase of the exhausted T cells expressing PD-1 correlated with patient prognosis (5).

Kong et al. have also shown that serum levels of 11 soluble checkpoints including GITR, 4-1BB, TIM-3, CD27, LAG-3, PD-1, CD28, CTLA-4, BTLA, HVEM, and CD80 correlated with severe illness in COVID-19 patients (30). Furthermore, patients with COVID-19 show increased Fas and PD-1 expressions in both CD4+ and CD8+ T cells. This also indicates the association of these regulatory molecules with the apoptosis of antigen-activated T cells during COVID-19, leading to decreased CD4+ T cell numbers and lowering the percentage of naive T cells (31). On the other hand, an elevation in the number of cells such as monocytes, neutrophils and natural killer (NK) cells causing cytokine storm has been reported (32, 33). Patients with more severe clinical conditions besides overexpressing proinflammatory cytokines such as IL-6, IL-1 and TNF-α, also display high levels of PD-L1 in monocytes and DCs. In the present work, we show that increased levels of sPD-L1 are associated with PD-L1 dysregulation in both epithelial and immune cells (34). In particular, epithelial cells permissive to SARS-CoV-2 upregulated PD-L1 expression. Induced PD-L1 expression can be restored by treatment with ruxolitinib, a JAK1 and JAK2 kinase inhibitor. Therefore, we hypothesize that JAK1 or JAK2 are involved in PD-L1 upregulation following SARS-CoV-2 infection in epithelial cells. However, mechanisms of PD-L1 upregulation in epithelial cells by SARS-CoV-2 should be further investigated. In vivo PD-L1 upregulation on infected cells might also reflect an increased cytokine release by the activated host immune cells. In both cases, PD-L1 upregulation provides infected cells with an immune escape mechanism to both innate and adaptive immune response facilitating viral replication and immunosuppression.

In addition, we have shown that PD-L1 is dysregulated on many types of immune cells of COVID-19 patients. Dysregulation of the PD-L1 gene correlates with substantial phenotypic differences between COVID-19 cases and controls, predominantly in monocytes, gamma delta T cells, neutrophils and CD4+ interferon-stimulated T cells. Several innate immune cell subsets are depleted in COVID-19 patients, including gamma delta T cells, DCs, plasmacytoid DCs, CD16+ monocytes and NK cells. Monocyte rearrangement is likely to reflect the elevated IL-10 levels observed in COVID-19 patients. IL-10, as an inhibitory cytokine, prevents T-cell generation and thus disrupts and reduces T-cell activation and proliferation, leading to dysfunction of cellular immune responses (35). Furthermore, along SARS-CoV-2 induced viral infection, signal transducer and activator of IL-10 secretion led to overexpression of PD-1 and PD-L1 in monocytes and DCs (36). It has been reported that monocytes involved in COVID-19, similar to monocytes involved in HCV, overexpress PD-L1 and IL-10. Noteworthy patients with more severe clinical states have higher expressions of PD-L1 on monocytes, DCs and granulocytes (37). Similarly, the attachment of PD-L1 from monocytes to PD-1 expressed on the surface of CD8+ T lymphocytes also inhibits their antiviral activity and ultimately leads to disease progression. Lastly, increased expression of PD-L1 on neutrophils and gamma delta T cells might also hamper their ability to eliminate infected cells. Gamma delta T cells do not recognize classical peptide antigens, their TCRs are non-MHC restricted, and they can respond to pathogen-associated molecular patterns and produce cytokines in the absence of TCR ligands. They can also defend against viral infection by secreting IFNγ and upregulating the expression of NKG2D, perforin, granzyme B and FasL. Following injury, resident cells release inflammatory cytokines and chemokines to recruit reparative neutrophils to the injury site. Excess inflammation, however, can result in undesired tissue damage. Therefore, the body’s ability to control inflammation is tightly regulated. PD-L1 expression on neutrophils increases with inflammation and correlates with impaired antibacterial function (10, 38). Even more PD-L1 overexpression on neutrophils correlates with markers of T cell exhaustion and contributes to suppression of T cell function resulting in an immunosuppressive activity in other types of viral infections such as HIV (39). Targeting PD-L1 with blocking antibodies can also enhance neutrophil innate immune function (40, 41). Lastly, decreased expression of PD-L1 on CD4+ interferon-stimulated T cells in COVID-19 patients may reflect the induction of a hyperactivation status which causes an excessive immunopathology (42).

Some data indicate that SARS-CoV-2 infection might persist in some tissue compartments. In individuals infected with HIV and on anti-retroviral therapy, immune checkpoint proteins themselves identify cells preferentially infected with HIV that persist on anti-retroviral therapy (43, 44). This observation is of great importance in efforts to eliminate residual virus that persists despite anti-retroviral therapy, as these infected cells are a major barrier to a cure. Whether PD-1/PD-L1 axis is involved in SARS-CoV-2 latency in long term SARS-CoV-2 infected patients should be further investigated.

## Conclusions

In conclusion, this study highlighted the potential role of PD-1/PD-L1 axis in COVID-19 and suggests a prognostic role of sPD-L1. These data have clinical significance since they provide a further rationale to implement novel clinical studies in treating COVID-19 patients with PD-1/PD-L1 inhibitors. Recent data in cancer patients treated with anti-PD-1 or anti-PD-L1 inhibitors have shown contrasting results on the safety and efficacy of these checkpoint inhibitors in protecting or exacerbating COVID-19 infection. Administration of checkpoint inhibitors such as anti-PD-1/PD-L1 should be investigated early in COVID-19 progression and especially should be avoided in critically ill patients where the immune system is already hyperactivated. Further studies will be needed to establish the potential timing of the administration of checkpoint inhibitors in COVID-19 patients. sPD-L1 as well as lymphopenia and elevated levels of CRP could be considered as biomarkers to identify and monitor patients who are likely to benefit from treatment with immune checkpoint inhibitors.

## Data Availability Statement

The raw data supporting the conclusions of this article will be made available by the authors, without undue reservation.

## Ethics Statement

The studies involving human participants were reviewed and approved by Comitato etico Campania Sud. The patients/participants provided their written informed consent to participate in this study.

## Author Contributions

Conception and design: FS and VC. Development of methodology: FS, VC, GF, and GS. Acquisition of data: CS, VM, AM, FAS, GG, and CZ. Analysis and interpretation of data: FS, VC, GF, PP, PZ, and SP. Writing, review, and/or revision of the manuscript: FS, VC, PP, and CC. Administrative, technical, or material support (i.e., reporting or organizing data, and constructing databases): AC, IP, EB, and MC. Study supervision: AF and SP. Other (contributed clinical and pathological material; discussed results and implications of findings): CC, CV, AF, and SP. All authors contributed to the article and approved the submitted version.

## Funding

The work was supported by Ministero dell’ Università e della Ricerca (Progetti di Rilevante Interesse Nazionale (PRIN), 2017, CODICE 2017PHRC8X_003) (to SP).

## Conflict of Interest

The authors declare that the research was conducted in the absence of any commercial or financial relationships that could be construed as a potential conflict of interest.

## Abbreviations

ARDS, Acute Respiratory Distress Syndrome; CRP, C-reactive protein; DAB, 3;3’ diaminobenzidine; ELISA, enzyme-linked immunosorbent assay; ESR, erythrocyte sedimentation rate; FFPE, Formalin-fixed paraffin-embedded; GEO, Gene Expression Ominibus; IHC, immunohistochemical; LDH, lactate dehydrogenase; LOS, length of stay; mAb, monoclonal antibody; min, minutes; OD, optical density; PaO_2_/FIO_2,_ arterial oxygen partial pressure/fractional inspired oxygen; PBMC, peripheral blood mononuclear cells; PD-1, programmed death-1; PD-L1, programmed death-ligand 1; sPD-L1, soluble PD-L1.
