# Supplementary material for: PD-L1 Dysregulation in COVID-19 Patients
Source: Front Immunol. 2021 Jun 7;12:695242. doi: 10.3389/fimmu.2021.695242 (PMC8215357; doi:10.3389/fimmu.2021.695242)
Supplement: Supplementary file 1 [file Table_1.docx]

**Supplementary Table 1. Details of patient comorbidities and levels of sPD-L1.**

| **Hypertension** | **Diabetes** | **Dyslipidemia** | **Cardiovascular disease** | **Immune disorder** | **Chronic pulmonary disease** | **Chronic kidney disease** | **sPD-L1** |
| --- | --- | --- | --- | --- | --- | --- | --- |
| - | + | - | + | - | - | - | 0.068175 |
| + | - | - | - | + | - | - | 0.1009 |
| + | - | - | - | - | - | - | 0.098923 |
| - | - | + | - | - | - | - | 0.076 |
| - | - | - | - | - | - | - | 0.122 |
| - | - | - | - | - | - | - | 0.308 |
| - | - | - | - | - | - | - | 0.402 |
| + | + | - | + | - | - | + | 0.133884 |
| + | + | + | + | - | - | - | 0.233368 |
| - | - | - | - | + | - | - | 0.111513 |
| - | + | + | - | - | - | - | 0.73 |
| - | - | - | - | - | + | - | 0.206 |
| - | - | - | - | - | - | - | 0.148 |
| - | - | + | + | - | - | - | 0.171 |
| - | - | - | - | - | - | - | 0.062115 |
| - | - | - | - | - | - | - | 0.099587 |
| + | - | - | - | + | + | + | 0.086936 |
| - | - | - | - | - | - | - | 0.065 |
| + | - | - | - | - | - | - | 0.269 |
| + | - | + | - | - | - | - | 0.113495 |
| + | - | - | - | - | - | - | 0.13781 |
| + | + | - | - | - | - | - | 0.121 |
| + | + | + | - | - | - | - | 0.116135 |
| + | - | - | - | - | - | - | 0.196 |
| - | + | - | - | - | - | - | 0.116 |
| + | - | - | - | - | + | - | 0.06 |
| + | + | - | - | - | - | - | 0.112174 |
| + | - | - | - | - | - | - | 0.077 |
| + | - | - | - | - | - | - | 0.08 |
| + | - | - | - | - | - | - | 0.182525 |
| - | - | - | - | - | - | - | 0.047916 |

**Supplementary Table 2. Details of patient treatment and levels of sPD-L1.**

| **Corticosteroids** | **Ruxolitinib** | **Eculizumab** | **Azytromicin** | **LMWH** | **Ceftriaxone** | **Tocilizumab** | **sPD-L1** |
| --- | --- | --- | --- | --- | --- | --- | --- |
| + | - | - | + | + | - | - | 0.068175 |
| + | - | - | + | - | + | - | 0.1009 |
| + | - | - | + | + | + | - | 0.098923 |
| + | - | - | + | + | - | - | 0.076 |
| + | - | - | + | + | + | - | 0.122 |
| - | - | - | + | + | - | - | 0.308 |
| + | - | - | + | + | + | - | 0.402 |
| + | - | - | + | + | - | - | 0.133884 |
| + | - | - | + | + | - | - | 0.233368 |
| + | - | - | + | + | + | - | 0.111513 |
| + | + | + | + | + | - | - | 0.73 |
| + | - | - | - | + | + | - | 0.206 |
| + | - | - | + | + | - | + | 0.148 |
| + | - | - | + | + | - | - | 0.171 |
| + | - | - | + | + | - | - | 0.062115 |
| + | - | - | + | + | - | - | 0.099587 |
| + | - | - | + | + | - | - | 0.086936 |
| - | - | - | - | - | - | - | 0.065 |
| + | - | - | + | + | + | - | 0.269 |
| + | - | - | + | + | - | - | 0.113495 |
| + | - | - | + | + | + | - | 0.13781 |
| + | + | + | + | + | - | - | 0.121 |
| + | - | - | + | + | - | - | 0.116135 |
| - | - | - | - | - | - | - | 0.196 |
| + | - | - | - | + | + | - | 0.116 |
| + | - | - | + | + | - | - | 0.06 |
| + | - | - | + | + | - | - | 0.112174 |
| + | - | - | + | + | - | - | 0.077 |
| + | - | - | - | + | - | - | 0.08 |
| + | - | - | + | + | - | - | 0.182525 |
| + | - | - | + | + | - | - | 0.047916 |
